# How to Become an Apomixis Model: The Multifaceted Case of *Paspalum*

**DOI:** 10.3390/genes11090974

**Published:** 2020-08-21

**Authors:** Juan Pablo A. Ortiz, Fulvio Pupilli, Carlos A. Acuña, Olivier Leblanc, Silvina C. Pessino

**Affiliations:** 1 Instituto de Investigaciones en Ciencias Agrarias de Rosario (IICAR), CONICET, Facultad de Ciencias Agrarias, Universidad Nacional de Rosario, S2125ZAA Zavalla, Argentina; ortiz@iicar-conicet.gob.ar; 2 Institute of Biosciences and Bioresources (IBBR-CNR), 06128 Perugia, Italy; fulvio.pupilli@ibbr.cnr.it; 3 Instituto de Botánica del Nordeste (IBONE), CONICET, Facultad de Ciencias Agrarias, Universidad Nacional del Nordeste, 3400 Corrientes, Argentina; caalac77@gmail.com; 4 UMR DIADE, IRD, Univ. Montpellier, 34090 Montpellier, France; olivier.leblanc@ird.fr

**Keywords:** agamospermy, plant breeding, plant development, plant reproduction

## Abstract

In the past decades, the grasses of the *Paspalum* genus have emerged as a versatile model allowing evolutionary, genetic, molecular, and developmental studies on apomixis as well as successful breeding applications. The rise of such an archetypal system progressed through integrative phases, which were essential to draw conclusions based on solid standards. Here, we review the steps adopted in *Paspalum* to establish the current body of knowledge on apomixis and provide model breeding programs for other agronomically important apomictic crops. In particular, we discuss the need for previous detailed cytoembryological and cytogenetic germplasm characterization; the establishment of sexual and apomictic materials of identical ploidy level; the development of segregating populations useful for inheritance analysis, positional mapping, and epigenetic control studies; the development of omics data resources; the identification of key molecular pathways via comparative gene expression studies; the accurate molecular characterization of genomic loci governing apomixis; the in-depth functional analysis of selected candidate genes in apomictic and model species; the successful building of a sexual/apomictic combined breeding scheme.

## 1. Introduction

Apomixis (asexual reproduction through seeds) [1] has long been seen as an unprecedented natural tool to maximize plant breeding, with potential wide impact on global farming systems [2]. In close developmental connection with sexuality, it functions as either a digressed or a parallel pathway, ending in the generation of clonal embryos of maternal origin within viable seeds [3]. Besides the importance of understanding this puzzling reproductive mode to advance the basic knowledge on reproduction, the combined use of sexuality and apomixis could accelerate the generation of novel improved plant varieties while dramatically diminishing the cost of the process, turning the notion of customized, locally-adapted hybrid crops adapted to every farm plot into reality [2]. The potential benefits of harnessing apomixis vary from full exploitation and permanent fixing of heterosis to seed distribution for crops actually propagated vegetatively like potatoes or strawberries [4]. However, the effective use of this trait in plant breeding requires full knowledge of the molecular switch allowing the transition from sexuality to clonal seed reproduction.

In the last 30 years, coordinated international efforts have led to the elucidation of major molecular actors controlling apomictic development [5]. The integration of these genes into interlaced functional pathways is currently under investigation, in the prospect of generating optimized biotechnological tools. With this aim, modulating the expression of some critical genes has allowed the rewiring of apomixis components in sexual plants [5] and even induced the production of clonal seeds in rice [6,7,8]. However, the stability of synthetic apomictic rice remains to be tested by natural selection and validated in field conditions. In any case, the molecular triggers of apomixis in natural agamic complexes (i.e., species composed of diploid sexual cytotypes and polyploid apomictic counterparts) remain unknown. Moreover, the harnessing of the trait into plant breeding acquired an entire new dimension under the proposal that apomixis and sexuality might be ancient polyphenic phenotypes, with both pathways represented in all plant species, although many lineages have lost the capacity to shift from one to the other [9]. This hypothesis implies that sexual species (like major crops) could become apomictic by restoring the lost natural switch between both phenotypes, provided that the asexual route remained operational. In order to identify the natural triggers of apomixis, extensive reproductive characterization should be conducted in a high number of taxa, to extend our knowledge on sexual and asexual reproduction coevolution. Up to date, although apomixis was reported in at least 78 of the 460 angiosperm families [10,11,12], only a few species have been characterized from a molecular perspective. Moreover, less than 10% of the >350,000 flowering plant species have been examined using cytoembryological techniques, which suggests that new members previously assumed to be sexual might be added to the apomicts list soon [9]. Thus, there is a need to complete the information available on the cytoembryological, developmental, and molecular aspects of apomixis through the scrutiny of previously uncharted species, a task that should be ideally carried out by scientists from different countries, who have access and are familiarized with unique local materials collected from plant populations growing in their natural habitats.

*Paspalum* L. is one of the few genera in which sexual and asexual reproductive behaviors have been characterized side by side for more than 50 years. During this period, many approaches proved to be unsuccessful, while others offered significant advances. In this review, we aim at presenting the rationale supporting the work carried out in this particular genus, and, based on our previous experience, proposing a series of advisable steps that could help to explore the molecular control of the trait in other species, through genetic, genomic, and/or breeding approaches. We expect to favor the development of other research projects, in order to boost the investigation of this biologically amazing and complex field.

## 2. Cytoembryological and Cytogenetic Germplasm Characterization

While apomixis and sexuality frequently coexist within a species, sometimes within a single individual or even a single ovule (facultative apomicts), some genotypes reproduce mainly by apomixis (obligate apomicts) or only by sexuality (full sexuals). There is no possible way to carry out solid molecular comparisons of the sexual and apomictic developmental pathways in the absence of previous knowledge on the extent of apomictic reproduction in the particular species/genotype under study. Moreover, the cytological (e.g., the level of ploidy and meiotic behavior) and the embryological (e.g., the apomictic type and the temporal developmental progression of the trait) backgrounds must be carefully examined in order to select the appropriate plant material/time frame to be compared.

To provide materials for research and breeding purposes, an extensive living collection of *Paspalum* species was settled at IBONE CONICET, Corrientes, Argentina [13]. Cytoembryological and cytological analyses of these materials, covering 72 out of the total 370 species included in the genus, showed that approximately 75% of them are polyploid [14]. Besides, 68% showed some potential for apomixis [14]. Cytoembryological examination revealed that sexuality is represented by the double fertilization of reduced female gametophytes (FGs) of the *Polygonum* type, typically composed of an egg apparatus (one egg cell and two synergid cells), a large binucleated central cell, and a mass of proliferating antipodal cells at the chalazal pole [15]. Meanwhile, apomictic reproduction corresponds mainly to the aposporous type, with apospory initials (AIs) differentiating from companion nucellar cells and dividing mitotically to form non-reduced ESs, which occasionally coexist with a meiotic ES in the same ovule [16]. The *Paspalum* mature aposporous ESs usually contain an egg cell, one or two synergid cells, a binucleate central cell, and no antipodal cells [17,18,19,20]. *Hieracium*-type apospory (i.e., 8-nucleate non-reduced female gametophytes containing antipodal cells) was described in only two species: *Paspalum secans* Hitchc. and Chase [21] and *Paspalum simplex* Morong [22,23]. Moreover, *Taraxacum*-type diplospory (i.e., 8-nucleate non-reduced female gametophytes containing antipodals) was reported in three species: *Paspalum commersonii* Lam. (2n = 6x), *Paspalum longifolium* Roxb. (2n = 4x) and *Paspalum conjugatum* Berg. (2n = 4x) [24,25]. Finally, both aposporous and diplosporous types were detected together in *Paspalum minus* E. Fourn. [26] and *Paspalum scrobiculatum* L. [24]. 

In polyploid apomictic individuals, microsporogenesis is characterized by a statistically significant increase of asynapsis/desynapsis, lagged chromosomes, chromosome bridges, and micronuclei [16]. Such abnormalities were attributed to genetic rearrangements such as inversions or translocations affecting one particular chromosome [27,28]. Pagliarini et al. [29] reported the formation of non-reduced pollen in polyploid Brazilian accessions. Besides, the occurrence of restitution nuclei as a consequence of irregular or arrested meiosis has been associated with apomixis in *P. secans* [30], *P. conjugatum* [25], and *P. minus* [26]. 

All apomictic *Paspalum* species characterized so far are pseudogamous, i.e., they require the fertilization of the unreduced polar nuclei by a reduced male gamete to form the endosperm and a viable seed. The number of polar nuclei involved in pseudogamy is of practical importance in apomixis research, particularly when the mode of reproduction is determined by the flow cytometric seed screen (FCSS) method [31]. This methodology allows the identification of the origin of a seed (apomixis or sexuality) in large numbers of individuals by comparing embryo:endosperm DNA content ratios. While sexuality results in a 2:3 ratio, the outcome of apomixis usually differs. For instance, apomictic seeds of tetraploid pseudogamous *Paspalum notatum* show a 2:5 embryo:endosperm ratio (4x embryo and 4x + 4x + 2x = 10x endosperm). This method was successful during the last two decades for determining reproductive behaviors in individual or bulked seeds of several *Paspalum* species [23,32,33,34,35,36,37,38]. Another distinctive feature of seed development in *Paspalum* apomicts is the lack of deleterious response to deviations from the 2:1 maternal-to-paternal genomic ratio in the endosperm strictly required for sexual reproduction [39]. Typically, apomictic individuals form seeds with parental contributions showing a maternal genomic excess in the endosperm, e.g., 4:1 (8x:2x) in tetraploid apomicts [15]. 

Moreover, the study of the relationship between apomixis and ploidy is of importance when characterizing the biological materials to be used in apomixis research. For instance, according to the data reviewed by Ortiz et al. [14], 27 (37.5%) from 72 characterized *Paspalum* species showed multiple cytotypes with variable ploidy levels. Out of them, 22 (81.48%) displayed sexuality at lower ploidy levels and apomixis at higher ones. The exceptions were *Paspalum conspersum* Schrad. (only sexual individuals); *Paspalum distichum* L., *P. secans* (Itchc. & Chase), and *Paspalum proliferum* Arechav (only apomictic individuals); *P. scrobiculatum* L. (with sexuality reappearing at the highest ploidy level, i.e., 12x) [14]. Consequently, there is a positive yet not strict correlation between the increment of the ploidy level and the expression of apomixis. A flow cytometry estimation of the DNA content in the embryo and the endosperm in a seed-by-seed analysis of 77 *Paspalum* accessions allowed confirmation of the reproductive mode/ploidy level for several of the above-mentioned species and provided new information on the reproductive mode for 12 additional ones and one botanical variety [40]. Most apomictic *Paspalum* entities belong to multiploid species of autoploid origin. Each multiploid contains a sexual self-sterile diploid cytotype and a series of aposporous apomictic autopolyploid cytotypes, usually from 3x to 6x, with tetraploids as the most common cytotype. The two main *Paspalum* species used as models for apomixis research, *P. notatum* and *P. simplex*, form agamic complexes made up of diploid sexual and autopolyploid apomictic individuals [14].

Once a significant number of materials were subjected to cytogenetic and cytoembryological examinations, the next step to be followed is the selection of those species/genotypes in which sexual and apomictic development will be analyzed from the genetic and molecular points of view. In the case of *Paspalum*, the species that received most attention are *P. simplex*, *P. notatum*, *Paspalum rufum,* and *Paspalum guenoarum*. All of them grow as wild native populations in the argentine Litoral region, located within a limited radius around several local laboratories devoted to *Paspalum* apomixis research, which eases the collection and classification of novel germplasm. The genetic structure of some of these populations was carefully characterized, and sexual diploid individuals were proposed as the main genetic variability source, since they co-habit and hybridize with polyploid individuals [34,41,42]. Additionally, the selected species show sizes of ovaries and anthers that allow a relatively simple dissection with scalpels and forceps under a magnifying spectroscope when conducting experimental crosses. Moreover, they (as most *Paspalum* species) are perennial and flourish during the warm season, are well represented in rangelands used for cattle production systems, and cultivated within and without the natural distribution region to be used as forage, turf, and cereal [13]. Finally, besides the necessary considerations on germplasm availability, plant anatomy, life cycle, and agronomic potential, the species choice criteria might include the size of the genome. In this regard, the 1C DNA content values of the selected *Paspalum* species are among the smallest within the Poaceae tribe, ranging from 0.550 pg in *P. notatum* to 0.900 pg in *P. guenoarum* [40].

Chiefly among the points deserving consideration in the selection of the species for molecular analysis is the availability of natural or induced materials of identical ploidy and contrasting reproductive modes. These resources are essential for the subsequent establishment of useful populations segregating for apomixis, which will be used in inheritance analysis, genetic/epigenetic mapping, omics surveys, and breeding. Moreover, the comparison of sexual and apomictic developmental pathways using genotypes of different ploidy levels is directly not advised, simply because ploidy may affect expression levels independently of reproductive behavior at numerous loci [43,44]. Regarding this, a preliminary gene expression comparison between a *P. notatum* sexual diploid genotype and a newly formed sexual autotetraploid derivative revealed that at least 0.49% of the pre-meiotic floral transcripts changed their relative expression after an increment of ploidy [45]. According to these numbers and considering the existence of near 70,000 transcripts expressed in *Paspalum* flowers, as was revealed recently from RNAseq experiments [46], ploidy-related differential expression would involve around 3500 transcripts, a quantity equivalent to that detected in the sexual vs. apomictic reproduction comparisons [46]. These estimations agree with results reported by De Oliveira et al. [47], who selected 28,969 transcripts that were common to 2x sexual, 4x sexual, and 4x apomictic genotypes, and found 1173 of them differentially expressed between 2x sexual vs. 4x sexual plants, while 1317 were contrastingly represented between 4x apomictic and 4x sexual plants. This confirmed that ploidy-related polymorphic expression might severely interfere the unequivocal identification of transcripts related to the reproductive mode.

In several species of *Paspalum*, apomixis occurs mainly at polyploid levels and sexual counterparts of the same ploidy are not available in nature. Therefore, colchicine treatments were required to duplicate the chromosome content of sexual diploids [33,48,49,50,51,52]. Alternatively, colchicine-induced facultative apomictic polyploids were crossed to apomicts in order to produce full sexual descendants [53]. Comparative genetic characterization of these recent artificial polyploids and the original diploids in *Paspalum plicatulum* and *P. notatum* revealed that, immediately after polyploidization, genetic rearrangements affected 28–38% and 9.55% of the genome, respectively [54,55]. Moreover, ancestral alleles lost after polyploidization were spontaneously recovered in further generations, a phenomenon previously reported in species of other genera [55]. Once the alleged sexual polyploids were obtained at the laboratory, their reproductive phenotypes might be controlled periodically, since one cannot rule out that plants showing high levels of sexuality might express some capacity for apomixis in a different environmental/seasonal condition (and vice versa), as described for *Eragrostis curvula* [56] and *P. notatum* [36]. It should be noted that sexual mother plants used to produce families segregating for the reproductive mode are expected to lack the genomic region/s determining apomixis and, therefore, any capacity for asexual reproduction. Artificial sexual materials of the proper ploidy must be repeatedly and extensively checked by cytoembryological and/or molecular progeny tests [50,53,57].

Finally, comparative transcriptomic and epigenetic surveys require the establishment of detailed timelines on reproductive development (i.e., timeframes in which the subsequent reproductive developmental stages were unambiguously demarked), to maximize the discovery of true differential representation results. Several reproductive calendars were established by correlating morphological features, e.g., ovary/ovule size and morphology, as well as mega- and microgametophyte developmental stages in *P. simplex* [49], *P. notatum* [58], and *P. rufum* [59].

**Figure 1:**
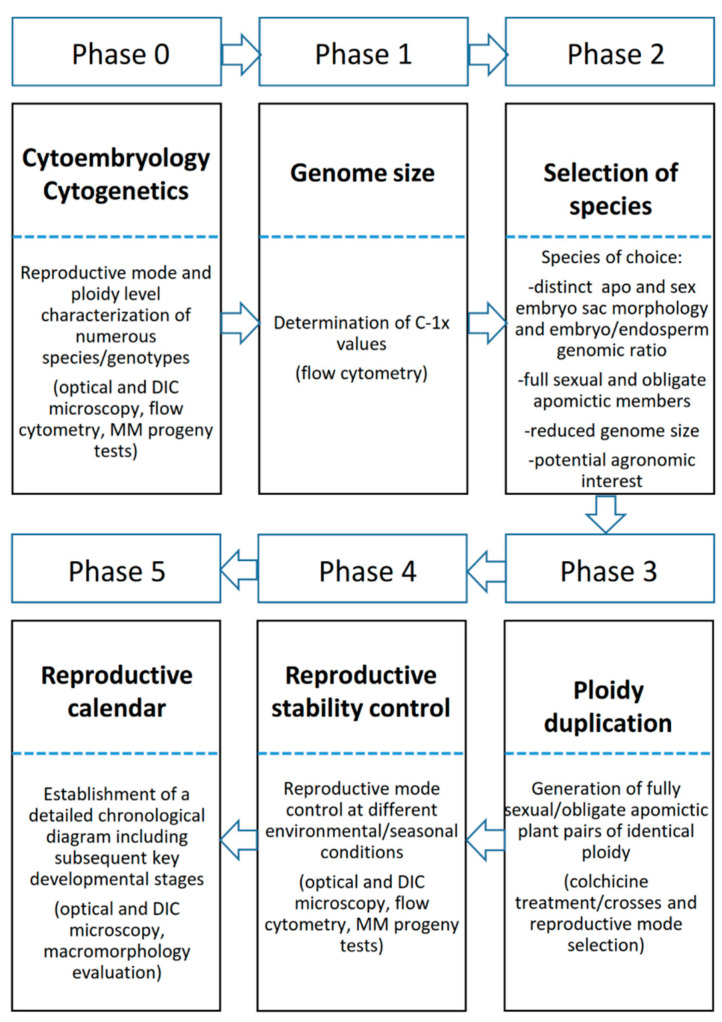
Consecutive phases of plant material preparation. In a clockwise sense, the series of phases that should be considered to produce germplasm suitable for apomixis molecular characterization. Methods/techniques are listed at the bottom of each column, within brackets. MM: molecular markers.

In summary, as schematized in Figure 1, preliminary germplasm characterization includes a series of successive obligatory steps leading to the selection/generation of plants with appropriate reproductive phenotype/genetic backgrounds to be used in genetic mapping, (epi)genomic and transcriptomic assays, together with the setting of precise time frames to standardize the collection of RNA/DNA samples.

## 3. The Genetic Control of Apomixis in *Paspalum*

Understanding and exploiting the advantages apomixis represents for plant breeding requires detailed knowledge of the genetic and molecular bases that control both its inheritance and expressivity. Although genes governing specific reproductive steps have already been engineered into plants and artificial systems can mimic clonal reproduction [60,61,62,63,64,65], the whole apomictic program has not been reproduced in sexual models yet [9]. This is the consequence of a complex genetic and/or epigenetic control of the trait, far more intricate than initial inheritance studies had suggested. 

In *Paspalum*, inheritance analyses were unfeasible until fully sexual tetraploid individuals were available [14]. Indeed, initial attempts suggesting a control by a few recessive genes [48] could not be further confirmed [66]. Towards the end of the 90s, however, sexual tetraploid individuals were experimentally generated for *P. notatum* [57], *P. simplex* [49] and, more recently, for *P. plicatulum* [33]. These genotypes became the female progenitors of segregating populations used in apomixis inheritance genetic dissection and breeding programs. Currently, a large number of well-characterized sexual tetraploid individuals, generated by chromosome doubling treatments (sometimes followed by experimental crosses) are available for the three species [33,50,51,53,67,68]. Moreover, several sexual genotypes of *P. simplex* and *P. plicatulum* were also used as female parents in interspecific crosses with different members of the *Anachyris* subgenus and the Plicatula group, respectively, widening the genetic studies to other species of the genus [60,61,62,63,64,65,66,67,68,69,70].

Genetic analyses of F_1_ and BC_1_ *P. notatum* progenies showed that apospory is inherited as a simple dominant trait (Aaaa). However, distorted segregations were often observed in favor of sexuality (aaaa) with apomixis:sexuality ratios varying from 1:1 (expected) up to 1:9 [27,71,72,73,74,75]. In the case of *P. simplex*, the segregation evidence indicates that apomixis is under the control of a single dominant allele, whose transmission is biased towards sexuality, while mapping reveals an association between the Apomixis Controlling Locus (ACL) and the telomeric region of the long arm of rice chromosome 12 [23,76]. Inheritance studies in species of the Plicatula group using F_1_ and F_2_ generations and three backcross populations derived from an artificial tetraploid sexual clone of *P. plicatulum* and an apomictic *P. guenoarum* cv. Rojas plant confirmed the previous genetic model, with a single Mendelian dominant factor with altered transmission (1 apo:1.6 sex) [67]. Interestingly, high rates of distortion against apomixis were also found in interspecific crosses involving *P. simplex* x *Paspalum malacophyllum* (1:5) [77] and *P. simplex* x *Paspalum procurrens* (1:15.7) [69]. Moreover, distortion of segregation has been reported in several grass and non-grass apomictic systems [78]. The large chromosome rearrangement (inversion or translocation) associated with the apomixis locus reported by Podio et al. [28] is to date the most relevant evidence to provide a mechanistic explanation for this phenomenon in *P. notatum*.

All genetic analyses in *Paspalum* showed a strict co-segregation between apospory, parthenogenesis, and unbalanced endosperm formation, suggesting that their relevant genetic determinants are located in the same low recombining chromosome area [23,67,79]. The parthenogenesis of reduced egg cells in the genus was never documented. Moreover, although sexual seeds require a strict 2:1 maternal:paternal genome contribution to form the endosperm, viable seeds from apomicts usually form despite unbalanced parental genomic contributions [15]. 

Another central topic demanding special attention is the transmission of the apomixis expressivity capacity. Usually, apomictic individuals are classified as obligate when almost all ovules (>90%) show one or several aposporous embryo sacs (AESs), or facultative, when at least some ovules bear meiotic or mixed (i.e., meiotic and aposporous) female gametophytes [14]. In *P. notatum*, the analysis of several segregating populations generated using an apomictic male progenitor revealed a high variability in the proportion of ovules bearing AESs among the hybrids [72,74]. In many cases, two well-differentiated groups of apomictic hybrids with low and high levels of apospory, respectively, were recovered from the progenies [74,75]. Moreover, only a small fraction (less than 10%) of them showed an apospory expressivity as high as that detected in the apomictic male progenitor, and this proportion decreased in subsequent crosses with sexual genotypes [72]. At least in *P. notatum*, apospory expressivity can also show seasonal variation, reaching its maximum during peak flowering (summer) and decreasing in fall [80]. A similar variation depending on the environmental conditions was reported in *Paspalum cromyorrhizon* [81]. Although the parental genetic distance positively correlates with the number of aposporous hybrids detected in the progeny (i.e., plants showing at least one ovule containing one or more AES, which also produce apomixis-associated molecular marker polymorphic bands), it does not display any association with the expressivity of apospory (i.e., the average proportion of ovules containing at least one non-reduced female gametophyte), suggesting a separate control for these two reproductive components [75]. The above-mentioned body of evidence suggests that genetic or epigenetic factors may be affecting the expressivity of the trait. Moreover, embryo parthenogenesis and endosperm development capacities are both variable: plants with well-developed AES in >90% of ovules cannot produce viable apomictic seeds [82]. Parthenogenesis seems to be under epigenetic control in *P. simplex*, as artificial wide-genome demethylation significantly reduced parthenogenesis but had no effect on apospory [83]. Therefore, the formation of viable apomictic seed relies not only on the presence of the ACL, but also on as-yet unknown modifiers potentially affecting apomeiosis, parthenogenesis, endosperm development, and germination. In this regard, a precise evaluation of the apomixis capacity for a given material entails the use of a combination of experimental approaches, including mature female gametophyte examination (apospory capacity), flow cytometry (seed development), and progeny tests (germination and establishment of apomixis-derived progenies) [13]. 

It is a well-known fact that many *Paspalum* species form multiploid complexes composed of self-sterile sexual diploid and self-fertile pseudogamous apomictic polyploid cytotypes [14]. However, this general rule is challenged by several observations in *P. notatum* and *P. rufum,* including aposporous-like embryo sacs (AES-like) reported at the diploid level [32,50,84]; seed formation from aposporous sacs of diploid cytotypes pollinated using tetraploid progenitors [32,52]; artificial apomictic tetraploid plants emerging from colchicine-induced chromosome duplication in sexual diploid individuals [50,52]; increased apospory expressivity at the diploid level after hybridization [52]. Moreover, a SCAR marker derived from the *P. notatum* ACL was detected in the genome of 10 out of 57 diploid plants from a natural population (Juan Pablo A. Ortiz, personal communication). These results support the hypothesis that the factor/s controlling apomixis might occasionally occur in diploid individuals, but they remain silent until they are activated in response to hybridization and/or polyploidization.

**Figure 2:**
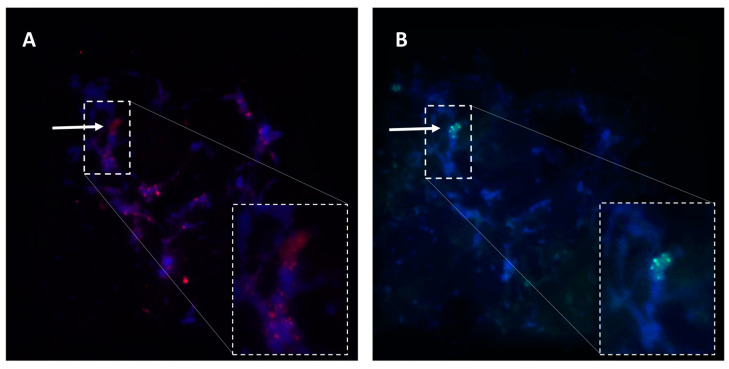
Methylcytosine immunodetection (**A**) and Apomixis Controlling Locus (ACL)-specific bacterial artificial chromosome fluorescence in situ hybridization (BAC-FISH) (**B**) on 4′,6-diamidino-2-phenylindole (DAPI)-stained pachytene chromosomes of apomictic *P. notatum* genotype Q4117. Arrows indicate an immunodetected heterochromatin knob (red dots) (A) and the ACL as revealed by BAC-FISH (green dots) (B) located at the same position. The region of interest was increased in order to show the details (bottom right corner). Maricel Podio (IICAR, CONICET-UNR, Argentina) kindly provided the images.

The *Paspalum* ACL, although quite simple in genetic terms, might be physically complex at the molecular level, as it was exposed by associating it to grass model species markers of defined map positions [69,76,77,85,86]. The apomixis-governing region is characterized by recombination restriction, hemizygosity, and heavy cytosine methylation (Figure 2) [27,77,79,83,86]. Comparative mapping showed syntenic relationships between the apomixis loci of at least four *Paspalum* species (*P. simplex*, *P. notatum*, *P. procurrens* and *P. malacophyllum*) and a 6–10 Mb region of rice chromosome 12, as well as a segment of 10 Mb of rice chromosome 2 (for *P. notatum* only) [77,86]. Although the ACL was conserved over generations within single species, small variations (short indels) were detected among species of the same section. More consistent changes occurred among species of different sections. In particular, members of the *Anachyris* subgenus show macrosynteny with the above-mentioned segment of rice chromosome 12 [69,77], whereas the ACL of *P. notatum* is a hybrid syntenic group resembling segments of chromosomes 12 and 2 of rice [77,86]. 

**Figure 3:**
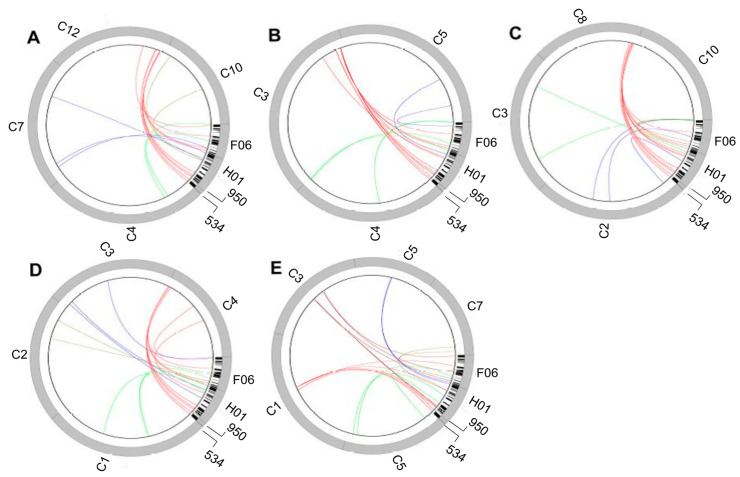
Mapping of genes located in four apomixis-linked BACs onto reference grass genomes, including *Oryza sativa* (**A**), *Setaria italica* (**B**), *Sorghum bicolor* (**C**), *Brachypodium distachyon* (**D**), and *Zea mays* (**E**). F06, H01, 950, and 534 correspond to contigs PS127F6_c1, PS366H1_c1, H10_950, and H10_534, respectively. C1-C12 represent chromosome numbers for each reference grass genome. Red lines link genes to the conserved chromosome area related to apomixis. Dr. Giulio Galla (DAFNAE, University of Padua, Italy) kindly provided the image.

A deeper analysis of the ACL structure arose from the availability of apomixis-specific *P. simplex* bacterial artificial chromosomes (BACs) isolated with the aid of specific primers. Partial sequencing of one of them, corresponding to the ACL, revealed that although synteny at the marker level was conserved with respect to the rice genome, gene micro-collinearity was frequently interrupted with transposable elements and migrant genes from other rice chromosomes [87]. More recently, Galla et al. [88] sequenced two other apomixis-linked BACs and discovered that the region of synteny of the *P. simplex* ACL is conserved among five reference grass species, being located at a telomeric position in chromosomes 12, 8, 3, and 4 of rice, *Sorghum*, *Setaria,* and *Brachypodium*, respectively, and in a more centromeric region of maize chromosome 1 (Figure 3). Based on these findings, it was hypothesized that the ACL of *Paspalum* could have originated from an ancestral unstable genome region in which (i) sex-related genes were grouped by gene migration during speciation, (ii) a polyploidization event (through an intermediate triploid bridge) locally induced further small-scale rearrangements that, in turn, generated local sequence divergence, lack of chromosome pairing, and recombination blocking. This non-recombinant segment favored the accumulation of mutations, since they could not be discarded by meiosis. This kind of evolutionary and structural organization may have consequences on gene content and expression. Indeed, Polegri et al. [89] noticed that apomixis-linked genes tend to be expressed in a constitutive mode throughout reproductive development. Moreover, other apomixis linked genes are specifically expressed in germ line committed cells, i.e., nucellus cells originating apospory initials, polar nuclei, and egg cells [88,90]. 

The expression of the ACL genes seems to have common features with operon-like gene clusters, which are defined as a set of two or more non-homologous functionally related genes that share a close genomic neighborhood [91]. Operons in plants probably originated by an initial event of gene duplication followed by neo-functionalization [92]. Similarly to the ACL, the operon like gene clusters originated from subtelomeric dynamic regions and are characterized by high rates of gene rearrangements [93]. Another multigene complex that shows striking similarity with the ACL of *Paspalum* is that related to the Y-chromosome of dioecious plants, which originated from autosomal chromosomes by initial suppression of recombination in the regions containing the sex controlling genes and later on, by the migration of male determining genes [94]. From a functional point of view, the evolution of the Y-chromosome induces both the silencing of the female genes, and the development of male function by the action of specific genes [94]. This finding could support the hypothesis of apomixis silencing key genes of sexuality. Podio et al. [83] showed that parthenogenesis in *P. simplex* is superimposed over fertilization-mediated embryo development by a mechanism controlled by DNA methylation. Furthermore, several *P. simplex* apomixis-linked genes expressed sense and antisense transcripts in reproductively committed cells, and showed a putative silencing effect of the apomixis-linked alleles on their sexual-specific counterparts [88,90].

## 4. Identification of Candidate Genes through Transcriptome Comparisons

In addition to the identification of key triggering factors, our knowledge on apomixis should be complemented by the understanding of the molecular pathways involved in subsequent developmental steps. Although genetic analyses suggest that apomixis in *Paspalum* spp. is under the control of a single genomic region, the intrinsic characteristics of the ACL (i.e., absence of recombination within a large chromosomal segment and abundance of repetitive sequences) hampers the identification of the trait’s key determinants through positional mapping strategies. In this scenario, a combination of transcriptomic and genetic/genomic information could provide hints on the primary or secondary role of DETs and be the method of choice for identifying genes and molecular routes involved in both activation and progression of the trait. 

However, several concerns emerge for comparative transcriptomic analyses using apomictic/sexual systems: (i) the genetic nature of the materials, since comparisons usually involve polyploid, highly heterozygous individuals, which complicates the distinction between differential expression and genetic polymorphisms in PCR-based analyses as well as the classification of homologs (orthologs, paralogs) in RNAseq approaches, in particular if no genome sequence is available; (ii) the sample collection timing, because the rapid progression of the RNA landscape along with reproductive development might cause the frequent emergence of false DETs; (iii) the short- and long-term transcriptome responses after hybridization and polyploidization, since some of the materials used might have recently originated from colchicine treatments, while others emerged from natural whole genome duplication events in either ancient or relatively recent times [42]. Unraveling these major drawbacks requires consideration of way out strategies like, for instance, the use of bulked segregant analyses involving several apomictic and sexual segregating offsprings, differential expression validation in numerous individuals by qPCR and/or in situ hybridization, standardization of sample collection protocols, RNAseq technical and biological replication, as well as attenuation of circadian and environmental effects by collecting samples at defined daytimes and/or conditions. After identification of apomixis candidates, their genomic locations must be recognized, to determine if they are being transcribed from the ACL or anywhere else in the genome. In the first case, the candidate could potentially be one of the triggers of the apomictic pathway. Otherwise, it could be part of the downstream molecular cascade involved in asexual reproductive development. To unequivocally map the sequence of interest, relatively large family populations segregating for the mode of reproduction and/or a reference genome where the ACL has been located should be available.

Transcriptomic surveys carried out in *P. notatum* since the early 2000s produced a large list of genes whose expression correlate with the occurrence of key reproductive features. An initial comparative examination of bulked RNAs from spikelets of sexual and apomictic F_1_ hybrids at anthesis was carried out by differential display and led to the identification of one transcript (*ARP1*) encoding an KSP consensus domain previously detected in several *cdc* 2-regulated cytoskeletal proteins [95]. A second transcriptome analysis using the same methodology revealed 65 new transcripts differentially expressed at the premeiosis/meiosis stage [58], including members of the *LORELEI* family (*GAP1*) [58,96], the S-adenosyl-L-methionine-dependent methyltransferase family (*TRIMETHYLGUANOSINE SYNTHASE1*) [58,97]; the *MAP3K YODA* family (*QUI-GON JINN* [58,98] as well as several retrotransposons carrying transduplicated gene segments [58,99], sometimes involving apomixis-associated genes like *SOMATIC EMBRYOGENESIS RECEPTOR-LIKE KINASE* (*SERK*) [58,99,100], and long-noncoding transcripts possibly involved in splicing regulation [101]. Furthermore, differential expression analyses during early seed development (3–24 h after pollination) revealed ≈100 DETs possibly associated with the unbalanced genomic contribution found in pseudogamous endosperms (4:1), including transcripts related with transcription regulation, signal transduction (e.g., lectin-like protein kinase and CK2 protein kinase α 1), growth/division, and response to changes in the extracellular ATP levels [102,103]. 

In *P. simplex* cv. Morong, the use of cDNA-AFLP on RNA extracted from flowers at several developmental stages, from premeiosis to 3–6 days after anthesis, rendered 202 DETs, the majority of which were present in apomictic florets only [89]. Near 20 of them, mostly related to signal transduction and nucleic acid binding, mapped within the ACL and showed constitutive expression in apomictic plants. Interestingly, the majority of these sequences displayed nonsense and frameshift mutations, revealing a probable pseudogene nature [89]. The remaining transcripts, transcribed from non-ACL genomic regions, mostly showed regulatory and seed storage functions. Several of the *P. simplex* DETs belong to the same annotation classes of those reported for *P. notatum*, including extensins, YODA-like MAP3Ks, LRR-like proteins, transferase proteins, and retrotransposon proteins.

In the last decade, progress in DNA and RNA sequencing have provided biologists powerful tools to study and understand gene functions and interactions. However, the establishment of genomes/transcriptomes from genetically poorly characterized, polyploid, and highly heterozygous species might easily result in chimeric assemblies and/or fragmented transcripts [104,105,106]. Therefore, robust reference floral transcriptomes were initially assembled from sexual and apomictic tetraploid *P. notatum* spikelets by using the long-read Roche 454-FLX + technology [46]. Out of these reference transcriptomes (48,842 genes identified), a preliminary list of 3732 sexual vs. apomictic DETs was generated, revealing several molecular networks putatively altered during apomixis, mainly related to ribonucleotide metabolic processes, protein complex biogenesis and assembly, monosaccharide catabolism, translation, gene expression, proteolysis, protein transport, DNA replication, and regulation of RAS activity [46]. Since these reference transcriptomes were constructed from long reads (mean sequence length around 500 bp), they provided a solid frame to establish future short reads-based assemblies and allowed recovering of putative alleles/paralogs full sequences for thousands of genes. 

**Figure 4:**
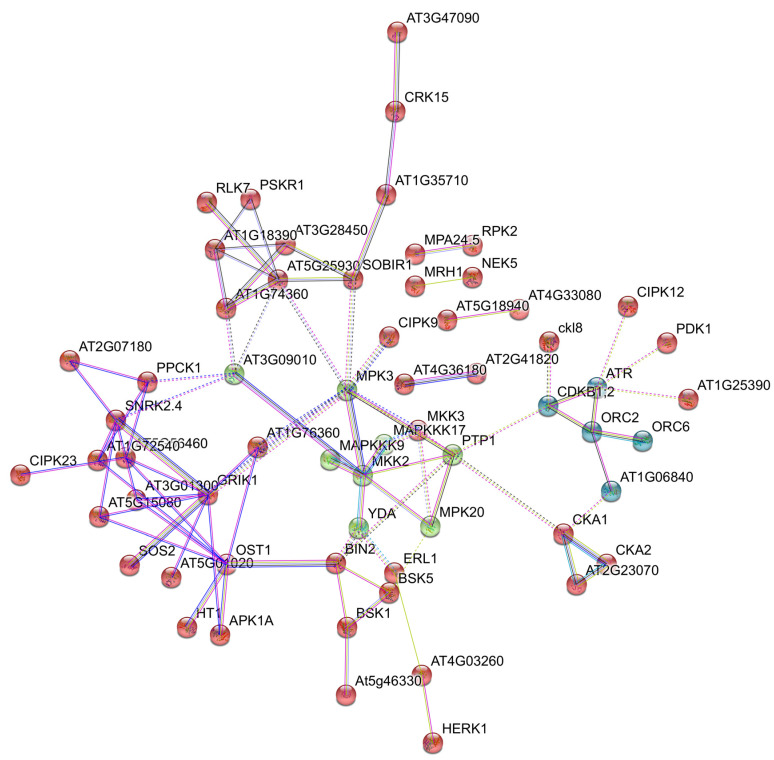
Gene network interactions controlling apomixis development. The scheme shows a particular group of apomixis-related predicted proteins and their functional interactions. The central node, MPK3 (mitogen-activated protein kinase 3), is encoded by the putative *Arabidopsis* ortholog to *QUI-GON JINN*, and was functionally related with apomixis by Mancini et al. [98] since its expression in the nucellus is necessary for aposporous embryo sacs (AESs) formation. MPK3 interacts with CIPK9 (CBL-interacting serine/threonine-protein kinase 9), a group of other MPK proteins (MKK2, MAPKKK17, MAPKKK9, and YDA), as well as to a homologous to AT1G76360 (a protein serine/threonine kinase) and AT5G25930 (protein kinase family protein with leucine-rich repeat domain). Nodes: query proteins and first shell of interactors with some known or predicted 3D structure. Edges: light blue: from curated databases; magenta: experimentally determined; green: gene neighborhood; red: gene fusions; blue: gene co-occurrence; yellow: text mining; black: coexpression; grey: protein homology.

While long-read Roche 454-FLX + sequencing produced genuine transcript assemblies, the derived quantitative expression comparisons lacked statistical accuracy (e.g., no technical replicas were established). Recently, De Oliveira et al. [47] reported a global gene expression analysis in *P. notatum* using Illumina sequencing of RNA samples extracted from leave and florets of 2x sexual, 4x sexual, and 4x apomictic genotypes. The database contains 114,306 reference transcripts, 536 of which correspond to genes possibly associated with apomixis. Interestingly, 89 differentially expressed transcripts mapped onto rice chromosome regions syntenic to the ACL [47]. Moreover, to provide a more comprehensive view of the sense/antisense transcriptomic landscapes emerging during reproduction in *P. notatum*, we generated Illumina TruSeq floral RNA libraries in triplicate, from sexual and apomictic materials collected at four different developmental stages, from premeiosis to anthesis (NCBI repository SRA accession: PRJNA511813). Data analysis (read assembly, comparative quantification, and predictive network interactions) is on-going. Figure 4 shows an example of a network possibly involved in apomixis, identified using the String v11 software [107].

Finally, the short-sequence components of the *P. notatum* sexual and apomictic floral transcriptomes (including siRNAs and miRNAs) and their possible target genes were characterized in detail [108]. A total of 1525 transcripts showed differential sRNA representation between sexual and apomictic plants, including genes related to meiosis, plant hormone signaling, biomolecules transport, transcription control, and cell cycle. Forty miRNAs precursors corresponding to conserved families and eight novel entities were identified. From them, 56 precursors showed miRNA differential representation between apomictic and sexual plants, always displaying upregulation in the apomictic sample. An analysis of the miRNA possible targets revealed 374 sequences, among which auxin-related genes were prevalent [108], opening a new line of research on the role of phytohormones in the switch from sexuality to apomixis.

## 5. Genomic Resources: The Lost Continent of Apomixis Research

As mentioned in previous sections, the mechanisms for establishing apomictic lineages from sexual ancestors and the evolutionary consequences of their emergence remain poorly understood and largely speculative. Mining the large body of knowledge gained in apomictic species, including *Paspalum*, points out hypotheses as diverse as polyploidization and hybridization [52,109,110], inactivation of epigenetic silencing pathways [111,112], oxidative stress during meiosis [113], functional trans-acting roles for a highly heterochromatic, hemizygous genomic region specific of apomictic plant genomes [90,98,114], miRNA deregulation [108,115], alterations in RNA splicing machinery [97], and hormonal signaling [116]. Although functional analyses in model species, including *Arabidopsis* and rice, have already provided valuable information regarding the role of some candidate genes, their positioning into a comprehensive genetic network controlling apomixis remains elusive to date.

Furthermore, apomictic plants usually have polyploid genomes, or possess aneuploid chromosome complements of highly heterozygous nature. Genetic approaches have revealed a singular genomic organization, characterized by a large, hemizygous and non-recombinant region of heterochromatic nature named Apomixis Controlling Region (ACR) in *Pennisetum* [78] or Apomixis Controlling Locus (ACL) in *Paspalum* [87]. Such genetic behavior and molecular features occur rarely in nature and, as mentioned before, are amazingly reminiscent of sex chromosome evolution, which involves the selection for suppression of recombination in a specific chromosomal region, followed by a massive rearrangement characterized by repetitive sequences accumulation, gene silencing, and loss of function [117,118,119]. Determining the genetic variation functionally relevant to build apomictic developments as well as the effects that in return may have shaped the architecture of apomictic genomes remain a key focus in apomixis research. This challenge requires the establishment of research platforms accommodating biological information and molecular resources for the genomes found in agamic complexes. Unfortunately, albeit advances in molecular and acid nucleic sequencing technologies and bioinformatics have widened genomics and transcriptomics approaches at an unprecedented scale [120,121], assembling the genome of an apomict remains a difficult task, because of complications rooted in their biology, as shown above.

To date, a few genomes of sexual relatives of apomictic plants have been sequenced, including *Boechera retrofracta* [122] and *E. curvula* [123], providing valuable information on genome evolution and relationships within agamic complexes. The vast amount of botanical, phylogenetical, cytoembryological, and transcriptomic information collected from numerous *Paspalum* species and agamic complexes [14,46,108] makes this genus a unique model system to explore the interplay between genome evolution and the emergence of apomictic reproduction in plants. *Paspalum* genomes are relatively small, as 2C values range from 0.5 to 6.5 pg [40]. Interestingly, in-depth characterization of the *Paspalum* ACL has revealed various extents of synteny with the subtelomeric part of the rice chromosome 12 long-arm [40,88], and shows structural features of heterochromatin [86,87]. Recently, we have initiated long-read sequencing and optical mapping approaches for generating genome assemblies of sexual and apomictic plants from several *Paspalum* species. Once assembled and annotated, we expect to molecularly resolve the ACL and determine its evolutionary trends; to re-analyze *Paspalum* transcriptomic resources, and to generate and compare epigenomes of sexual and apomictic plants. Finally, this effort will generate spillovers of great interest for forage grass breeding by providing critical genomic resources and allowing efficient molecular breeding, an important issue considering the need to mitigate both climatic change and anthropic pressures in economically important grassland and pasture agro-ecosystems.

## 6. Functional Analysis of Apomixis-Related Candidate Genes

After identifying candidate genes, the next rational step to advance apomixis research further consists of the establishment of functional analyses aimed at investigating their capacity to activate at least some steps of the trait. Those transcripts displaying differential regulation in apomictic/sexual backgrounds can be readily investigated through reverse genetic approaches, by using defective mutants or sense/antisense/RNAi transformants, since their down- or upregulation within the required genetic background would allegedly cause the emergence of apomixis-related phenotypes. Unfortunately, the absence of wide range germplasm banks for natural apomictic species forces either the prediction of orthology in model species or the adjustment of gene transformation techniques.

Based on these considerations, the steps to be followed when launching the functional characterization of an apomixis candidate gene in non-model apomictic/sexual systems like *Paspalum* are as follows: (1) previous spatio-temporal and sense/antisense expression validation of candidate genes with a protocol allowing cell-specific expression detection and distinction of the expressed RNA strand, like in situ hybridization; and (2) generation of overexpression/downregulation transformants with an efficient transformation platform, or identification of mutant germplasm involving model species orthologs. While the first step (spatio-temporal plus sense/antisense validation) will provide information useful for planning modulation (up- vs. downregulation), selecting the required reproductive background (sexual or apomictic) and guiding the promoter choice, the second one (mutant/transformant germplasm identification or generation) will facilitate the analysis of possible reproductive phenotype alterations. 

A point that deserves special attention is that, at least for many candidate genes, wide-range expression is associated with different functions in a multiplicity of organs/developmental stages, and only a subtle, timely, and cell-specific activity change might produce a deviation in the reproductive mode without causing collateral, sometimes detrimental, consequences. Therefore, when intending transformation, gene promoters of choice might be preferably time and organ/cell-type specific. Even if basic research could take advantages from additional information derived from constitutive promoter-based functional analysis, due to its potential to reveal the candidate function in numerous organs/tissues, the identification and effective use of cell-specific promoters is no doubt a bottleneck for apomixis-based breeding, which requires the modification of a restricted function set. If the project aims at generating a methodology to induce apomixis for breeding purposes, it should contemplate the identification, isolation, and/or validation of appropriate cell-specific promoters in the species of interest. On the contrary, when dealing with functional characterization of the gene, a constitutive promoter with verified expression in the ovule could be of choice, unless it causes unviable phenotypes due to deleterious side effects. 

**Figure 5:**
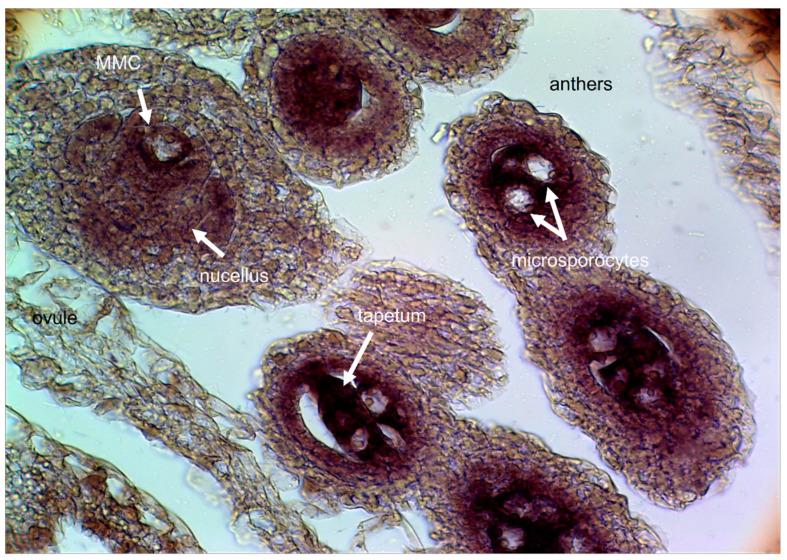
In situ hybridization of gene *ORC3* in reproductive tissues of *P. simplex*. Hybridization pattern of *PsORC3* (sense probe) in ovules and anthers of an apomictic genotype before the onset of meiosis. A strong hybridization signal is visible in nucellar and tapetum cells. The megaspore mother cell (MMC) does not express the target transcript. Dr. Lorena Siena (IICAR, CONICET-UNR, Argentina) kindly provided the image.

In connection with this, in situ RNA hybridization protocols were developed for *Paspalum*, and gradually optimized to analyze the spatio-temporal plus sense/antisense expression distribution within the ovule [38,58,90,96,97,98,99,100,101]. An example of expression characterization in reproductive tissues at early megasporogenesis involving the apomixis candidate gene ORC3 is shown in Figure 5.

**Figure 6:**
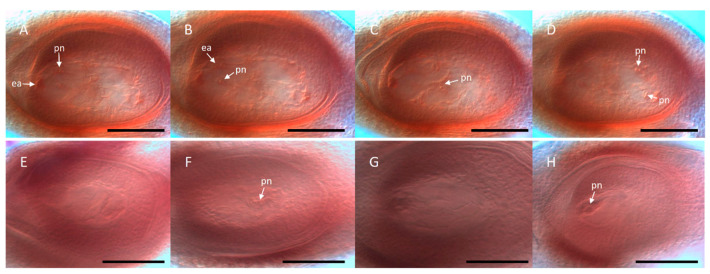
Cytoembryological analysis of RNAi lines with defective expression of the apospory candidate gene *QUI-GON JINN* (*QGJ*). The *QGJ* downregulation inhibits the formation of AESs in obligate apomictic plants [98]. (**A**–**D**) DIC consecutive focal planes of a single ovule originated from an obligate apomictic control, at anthesis, showing at least five typical supernumerary mature aposporous embryo sacs. (**E**–**H**) DIC single focal planes of a *QUI-GON JINN* defective RNAi line. Each image corresponds to a different ovary of the same plant, at anthesis. All of them (**E**–**H**) display abnormal morphology. pn: polar nuclei. ea: egg apparatus. Black bars: 100 µm.

Regarding the development of protocols for modulating the expression of candidate genes in natural apomictic systems, Mancini et al. [124] considered several previously developed methodologies [125,126,127] as the starting point to examine alternative explants/conditions for biolistic transformation, and designed a platform best suited to a wide range of *Paspalum* genotypes. Such methodology is currently being used to produce *Paspalum* lines with up- or downregulated expression of apomixis candidates, which are later subjected to reproductive phenotype analyses. An example of the cytoembryological characterization of an antisense transformant line with downregulated expression of the apospory-inducer candidate gene *QGJ* is shown in Figure 6. Moreover, the potential of cell-type specific *Paspalum* promoters identified from genome sequencing is currently under analysis (S. Pessino, unpublished). All these methods have allowed the functional characterization of three reproductive candidate genes (*ORC3*, *QGJ,* and *TGS1*) in species of *Paspalum* (see descriptions below), and several others are undergoing the same process.

### 6.1. PsORC3

A particular copy of the gene *ORC3* (*ORIGIN OF RECOGNITION COMPLEX 3*), identified as *PsORC3a*, resulted genetically linked to apomixis in all *Paspalum* spp. for which segregating populations were available [90,128]. In *P. simplex*, *ORC3* exists as three different copies, of which *PsORC3a* is a pseudogene specific for apomicts expressing an RNA transcript unlikely to be translated in a functional protein, whereas *PsORC3b*, probably coding for a highly conserved functional protein, together with *PsORC3c*, coding for a truncated protein, are common to both apomictic and sexual plants [90]. *PsORC3a* is poorly and constitutively expressed at all developmental stages in apomictic flowers only. In situ analysis showed that, in apomictic plants, sense and antisense strands of *PsORC3* are represented in cells and nuclei committed to reproduction (i.e., polar nuclei and egg cells), whereas both transcripts are silenced in the endosperm. Conversely, in sexual plants this gene is expressed as a sense transcript in the egg cell, polar nuclei, and endosperm, but not in the embryo [90]. Reverse genetics in both *Arabidopsis* and rice showed that *ORC3* defective genotypes display normal gametophyte development, but endosperm/embryo arrest at early stages of development [90]. Based on these considerations, we argued that the effect of the regulation of this gene on apomixis should be related to endosperm development. Particularly, we hypothesized that the apomixis-linked copy of *PsORC3* (*PsORC3a*) could be involved in a relaxation of control mechanisms, which allow endosperm development even facing a maternal genome contribution excess [90]. 

### 6.2. QGJ

The transcriptomic surveys carried out by Laspina et al. [58] identified a DET homologous to a mitogen-activated protein kinase kinase kinase gene (N46). Apomictic and sexual *P. notatum* Roche 454-FLX + floral transcriptomes were used to recover N46 full cDNA sequences and carry out a molecular phylogenetic analysis [98]. N46 was classified as a member of the *YODA* MAP3K family and renamed *QUI-GON JINN* (*QGJ*). At meiosis, in situ hybridization analysis revealed an altered pattern of expression in *P. notatum* apomictic plants, which could be analyzed at even more detail in apomictic *Brachiaria brizantha* [98]. While in sexual plants *QGJ* was strongly expressed at micropylar degenerating megaspores, in apomictic ones it showed activity within the enlarged meiocyte and the distal ovule nucellus, but was absent from the cell layer surrounding the meiocyte, from which apospory initials (AIs) originate. The effect of a *QGJ* diminished expression in an apomictic background was further investigated by producing RNAi lines [98]. Relatively high proportions of aborted ovaries, defects in both initiation and completion of AES formation, as well as a substantially lower number of AES per ovule were detected in two independent RNAi lines in comparison to both wild type and transformation control plants [98]. The proportion of ovules containing meiotic ES (MES) showed no statistical difference among the obligate apomictic wild type (3–8% MES according to Ortiz et al. [57]), the control, and the RNAi lines [98]. The conclusion emerging from these results was that the significant reduction of *QGJ* expression in an obligate apomictic background impaired the formation of AESs and, therefore, the expression of *QGJ* in distal nucellar cells is needed for aposporous development [98]. Genetic mapping analysis showed no evidence of a genetic link between *QGJ* and the ACL, but a long non-coding RNA partially related in sequence to *QGJ* (*LNC_QGJ*) cosegregated strictly with apomixis when mapped in a *P. notatum* segregating population [98]. Moreover, reverse-transcribed PCR experiments using *LNC_QGJ* specific primers, which were conducted in several apomictic and sexual *P. notatum* individuals, showed that *LNC_QGJ* is expressed only in apomictic plants. The existence of a functional link between the particular *QGJ* expression pattern detected in apomictic plants and a putative regulatory long non-coding *LNC_QGJ* activity operating from the ACL should be further investigated [98].

### 6.3. TGS1

After identification of an apomixis-associated DET (N69) homologous to a PRIP-interacting methyltransferase S-adenosyl domain protein by Laspina et al. [58], this candidate sequence was extended and confirmed to be a plant-specific *TRIMETHYLGUANOSINE SYNTHASE 1* (*TGS1*) gene, which was named *PN_TGS1-*like [97]. In facultative apomictic plants, *PN_TGS1-*like showed expression levels positively correlated with sexuality rates. Moreover, it displayed contrasting in situ hybridization patterns in apomictic and sexual plant ovules from premeiosis to anthesis [97], with higher expression in ovules (including nucellus, integuments, and reproductive lineage) of sexual plants throughout development, from premeiosis to maturity. Since the nucellus is the site of aposporous initials (AIs) differentiation, we proposed that *PN_TGS1-*like might be preventing the differentiation of apospory initials in sexual *P. notatum* plants [97]. Thereafter, a full-sexual *P. notatum* genotype was transformed with a *TGS1*-like antisense construction under a constitutive promoter, to obtain lines with a reduced transcript representation [38]. Antisense plants developed prominent trichomes on the adaxial leaf surface, occasionally formed twin ovules, and showed around 15% of ovules bearing what looked like supernumerary aposporous-like gametophytes (i.e., numerous female gametophytes with a typical *Paspalum* unreduced megagametophyte morphology, including an egg cell, one-two synergids, two polar nuclei, and no antipodal cells). Moreover, around 9% of ovules showed a combination of meiotic and aposporous-like sacs. At early developmental stages, 32% of ovules displayed nucellar cells with prominent nuclei resembling apospory initials (AIs) surrounding the megaspore mother cell (MMC) or the MMC-derived meiotic products. Occasionally, immature binucleated (FG2) female gametophytes of the aposporous type (i.e., the two nuclei located at the same side of the central vacuole) were detected [38]. Neither multiple meiosis nor early proembryos were registered, which suggested a non-reduced nature for the extra nuclei observed in the mature ovules and an absence of parthenogenesis, respectively. The antisense lines produced viable pollen and formed an equivalent full seed set after self-pollination. Flow cytometry analyses of caryopses revealed that all full seeds had originated from meiotic female gametophytes (i.e., by sexuality) supporting the hypothesis that parthenogenesis might not be operative [38]. Moreover, antisense lines showed a significant reduction of the germination percentage, indicating that *PN_TGS1-*like might also be involved in either embryogenesis or endosperm development. These results suggest that *PN_TGS1-*like is a developmental repressor, whose expression in leaves blocks the formation of trichomes, while in ovules inhibit the onset of apospory initials and/or the progression of gametophytes. However, it does not influence parthenogenesis, even when it might play an unknown role during embryogenesis.

## 7. Advances in Methods for Improving Apomictic *Paspalum* Species

The identification of molecular markers (MM) cosegregating with apomixis, the generation of artificial sexual polyploids after colchicine duplication, the construction of transcriptome databases and, in a close future, genomic assemblies, the identification of genes controlling the reproductive mode, and the establishment of biolistic transformation platforms offer good and innovative prospects for harnessing reproductive and non-reproductive traits of interest in *Paspalum* forage grasses, which have been selected and improved by classical and molecular methods over 80 years. The diversity present in the genus *Paspalum* for the modes of reproduction and ploidy levels is directly linked to adaptation to different environmental conditions and variation for a large group of traits of agronomic interest. Since the 1940s, near 30 apomictic cultivars belonging to the genus *Paspalum* have been released mainly for forage or turf [13]. All of these cultivars, except for one recently developed, are ecotypes collected in South America, evaluated in their target area, and used in different parts of the world, mainly in Australia, the United States, Japan, Thailand, Brazil, and Argentina. The success of this approach lies in the large diversity available in the genus and even within individual species, as reported for *P. simplex* [129]. However, a large number of desirable traits remain dispersed in the apomictic germplasm, among ecotypes and species, since genetic recombination is locked by apomixis [130].

The majority of the *Paspalum* species form agamic complexes [14]. The release of the genetic variability present in the apomictic germplasm was made possible after sexual tetraploid plants were generated by diploid plant chromosome duplication [33,48,49,50,51]. Since then, large segregating progenies have been generated by crossing these artificially induced sexual tetraploid genotypes as female parents and apomictic ecotypes as pollen donors. This procedure has been repeatedly used over the last years attempting to improve *P. notatum* [72,73,74,75] and, more recently, *P. simplex* [131] and species of the Plicatula group [70]. The experience with *P. notatum* indicates that only a reduced fraction of the progeny (around 10%) inherits the full capacity to express apomixis [72,73,74] and variable degrees of apospory expressivity are observed within hybrids [73,74,75]. Moreover, the self-incompatibility present in the diploid germplasm [132] and the induced sexual tetraploids is not transmitted to the sexual or apomictic progeny [72]. There is also evidence indicating that segregation for apomixis is independent of the segregation for traits of agronomic interest in *P. simplex* [131]. In the Plicatula group, several intra and interspecific crosses have been performed and different degrees of crossability and fertility of the resulting hybrids were detected [70]. Nevertheless, the possibility of hybridization is restricted because of flowering asynchrony among ecotypes and the sexual tetraploid germplasm. This issue may be overcome by storing the pollen of the apomictic ecotypes [133] or by creating new sexual tetraploid genotypes with different flowering times [70]. As a general rule for the genus, a large diversity is observed for agronomic traits as a result of crossing sexual and apomictic genotypes [13]. Heterosis for traits of interest, such as forage yield and cold tolerance, have been repeatedly reported in *P. notatum* [72,73,75,134] and for interspecific hybrids between *P. plicatulum* and *P. guenoarum* [70,135]. An apomictic hybrid of *P. notatum* identified as upright and fast-growing was recently released as a forage cultivar named Boyero UNNE, resulting from a collaborative research between the University of Florida and the National University of the North-East, Argentina [136]. 

MMs have been used to monitor the process of hybridization between sexual and apomictic genotypes in *Paspalum*, particularly for the identification of true hybrids within progeny [35,70,74,131]. The general idea is to test the progeny for the presence of male-specific MMs and for the segregation of female-specific markers indicative of recombination events in the female side. This procedure is particularly useful, since sexual tetraploid hybrids exhibit high levels of seed fertility when self-pollinated [72]. Moreover, several markers 100% linked to apospory have also been developed for the genus [77,85,137]. Some of these markers have been successfully applied to identify hybrids exhibiting apospory in *P. notatum* [74,75] and *P. simplex* [131]. Usually, they are useful for an initial evaluation that allows separating all the highly sexual progeny from the aposporous, but further analyses are needed to determine the different levels of apospory expressivity. This can be achieved by assessing phenotypic homogeneity in the progeny or by female gametophyte observations [74,75]. Moreover, random MMs are used in progeny tests, to determine the apomixis expressivity, and the genetic stability of an apomictic cultivar [136]. This is an important point to consider before going forward with the breeding process, because a relatively high expression of sexuality will reduce the stability of the new cultivar in a few years. An original alternative is to carry out gene expression analysis, by targeting those candidates whose activity is positively or negatively correlated with apomixis [97]. Although this approach can result much more complicated than cytoembryological analyses and progeny tests, it is expected to provide a good estimation of apomixis capacity and can be extended to high numbers of individuals. Finally, the lack of available apomixis-linked markers in many *Paspalum* species, including the Plicatula group, makes the use of cytometric seed analysis an attractive screening technique for determining the reproductive mode [138]. 

MMs have also been used to identify heterotic groups in *Paspalum*. Since the main objective of hybridization in apomictic species is to combine high levels of apomixis with heterosis for traits of agronomic interest, Marcón et al. [75] used ISSR and SSR markers to determine the genetic distances between a group of sexual and apomictic genotypes of *P. notatum*. Crosses were made between pairs of sexual and apomictic parents with low, intermediate, and high genetic distances. As expected, higher genetic distances between parents increased the proportion of hybrids exhibiting heterosis for forage yield. Moreover, the same positive relationship was observed between genetic distances and the proportion of aposporous hybrids identified within the progeny. However, apospory expressivity was not significantly related to the genetic distance. These results suggest that random MMs may be successfully used to determine heterotic groups for forage yield in *P. notatum*, but also to predict a high occurrence of apomictic hybrids within segregating families. Furthermore, as part of a hybridization project in the Plicatula group, the genetic distances between an induced sexual genotype of *P. plicatulum* and 22 accessions belonging to 12 different species were determined using AFLP markers [70]. In this case, the large variation for seed fertility and crossability was not correlated to the genetic distances. 

A synthetic sexual tetraploid population (SSTP) was established in *P. notatum* to increase the sexual tetraploid gene pool, as follows: (1) crossing the few available sexual tetraploid genotypes with a group of ecotypes collected across the Americas, and (2) polycrossing the resulting highly sexual hybrids to create the SSTP. This procedure allowed the transfer of the genetic diversity present among geographically and genetically distant ecotypes into a single sexual and cross-pollinated population [68,139]. More recently, a similar approach has been used to create a sexual tetraploid population in the Plicatula group [138]. In this case, nine accessions from six species with contrasting morphological characteristics were crossed to a single sexual induced genotype. The sexual hybrids were set apart using a cytometric seed screening and a group representing the different origins was polycrossed to obtain a large sexual population containing alleles from this diverse germplasm. This approach may also be used to generate sexual tetraploid populations for other species or groups of species in the genus sharing the same genetic characteristics, such as *P. simplex* or other species of the subgenus *Anachyris*.

The availability of sexual tetraploid populations has allowed the application of breeding methods originally developed for cross-pollinated crops, such as maize or alfalfa, as was originally suggested for apomictic tetraploid *Brachiaria* spp. [140]. Marcón et al. [141] evaluated the use of recurrent phenotypic selection (RPS) and recurrent selection based on combining ability (RSCA) in tetraploid *P. notatum*. Both methods proved to be efficient for improving forage yield in *P. notatum*. Although RSCA was expected to be more appropriate for exploiting heterosis since it was developed to accumulate heterotic effects over cycles, no difference was observed between the RPS and RSCA procedures for increasing forage yield [141]. A modified version of RSCA was used to identify superior tetraploid hybrids within the sexual population of Plicatula [138]. In this case, an initial phase of phenotypic selection was used to reduce the number of superior sexual hybrids, which were test-crossed to two elite apomictic clones belonging to *P. guenoarum*. This approach identified a few sexual hybrids as possible progenitors to continue tapping into genetic diversity of apomictic individuals or to generate improved sexual populations.

**Figure 7:**
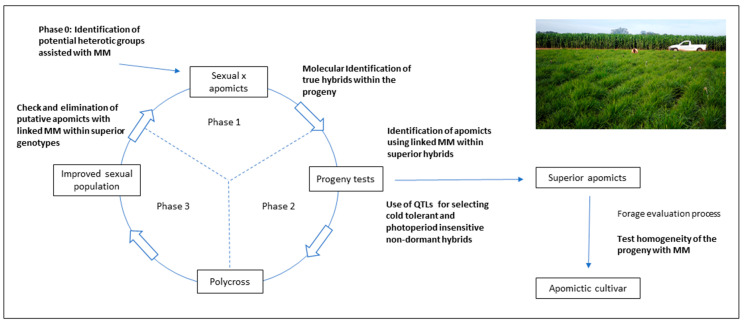
Theoretical scheme of recurrent phenotypic selection based on a combination of classical and molecular techniques in *P. notatum.* The molecular markers (MM) use scenarios are indicated at each phase of the process. An image of an experimental/seed production plot of an improved apomictic hybrid cultivar of *P. notatum* (cv. Boyero UNNE) is shown at top right.

As was previously stated, molecular techniques may play an important role in the different phases of the selection procedures for generating superior apomictic hybrids. A theoretical scheme of RSCA developed for tetraploid *P. notatum* is represented in Figure 7 to show how MMs may assist the breeding process. 

Random molecular markers may aid the identification of heterotic groups for the initial phase of the process (indicated as Phase 0 in Figure 7), as it was suggested by Marcón et al. [75]. Phase 1 consists of test-crossing a group of sexual genotypes to a single apomictic tester or a group of testers. When genetic data is expected to be collected, the true hybrids can be identified by using male-specific markers. The evaluation of the resulting progeny is indicated as Phase 2. The use of QTLs associated with traits of agronomic interest may be applied for the rapid identification of superior progeny. For instance, development of QTLs for cold tolerance and photoperiod insensitivity is underway for tetraploid *P. notatum,* since growth and forage production is markedly reduced during the winter in the subtropics. Once phenotypic and molecular data is available for selecting the best progeny, the apomictic genotypes may be separated using apomixis-linked markers. Superior apomictic hybrids usually need to go through a multiphase forage evaluation procedure [142]. The genetic homogeneity of the progeny coming from each selected line can be tested with random markers [136], to ensure the high expressivity of apomixis among the best lines. Phase 2 allows the selection of the best sexual genotypes that were crossed in phase 1, and these are polycrossed to generate an improved sexual population (phase 3 in Figure 7). This population is evaluated under field conditions, and only a few genotypes enter phase 1 of a new cycle. It is very important to check the mode of reproduction of this group of selected genotypes, and this can be done using apomixis-linked markers.

## 8. Conclusions

Given the dramatic increase in the world population, which is expected to reach 9.7 billion by 2050, and the concomitant expanding food demand, élite resilient crops should be readily developed on a massive scale in order to address the environmental, climate and overcrowding challenges [143]. Current methods used to produce new plant varieties, which rely entirely in the exploitation of sexuality, involve 7–20 years to bring them to the market, cost even millions of euros, and are limited regarding the harnessing of favorable gene interactions. Apomixis breeding could rapidly overcome the drawbacks associated with sexuality-based programs, facilitating a better adaptation to local environments, the avoidance of monocultures, and substantial increases in crop resilience [143]. However, specific methodological hitches complicate the generation and interpretation of the apomixis-related available molecular data and large areas of knowledge have barely been touched upon. To begin with, appropriate criteria should be established in order to reduce the number of candidate genes emerging from genomic and comparative transcriptomic surveys, based on both the combination of positional evidence with expression data and the establishment of reliable network predictions. Moreover, until now, no comprehensive study on the reproductive developmental impact of novel apomixis-related mRNA splice variants is available and protein post-translational regulatory mechanisms possibly influencing the trait remain uncharted. Finally, the regulatory role of transposons and lncRNAs was only poorly outlined and needs further clarification. The functional analysis of these aspects, once implemented, in combination with data body already available (Nanopore-derived genome and Roche 454-FLX +/Illumina transcriptome sequence databases) will lead to the application of accurate genetic engineering approaches by using sense or antisense transgenesis, RNA-directed DNA methylation, CRISPR-Cas9 editing and other techniques and allow the complete harnessing of the trait into plant breeding by inducing apomixis in sexual plants. 

From the *Paspalum* example we can derive the complex series of steps necessary to produce useful and applicable knowledge in apomictic species, consisting of (1) germplasm groundwork, including collection of variable materials, reproductive phenotyping, ploidy level assessment, genome content characterization, ploidy manipulation and reproductive calendar construction; (2) inheritance analysis, dense genetic mapping, and identification of markers cosegregating with apomixis; (3) genome sequencing; (4) transcriptome sequencing; (5) functional analysis in sexual and apomictic species; (6) traditional and molecular marker-assisted breeding to release élite apomictic hybrids. The molecular elucidation of the sexual–asexual transition would require the use of this methodological scheme in as many species as possible, in order to expose subtle variants that might have emerged during evolution and provide a comprehensive perspective of alternative pathways, disclosing optimal tools for harnessing apomixis into the breeding of major crops by genetic engineering. With this review, we hope to contribute to the design of the necessary experimental phases, which have been laid down in *Paspalum* after decades of effort, in a process that often involved trial and error. At best, the ordered management of the flowchart presented here could save both time and costs and stimulate apomixis research/breeding in other species of agronomic interest.
